# Optimizing Router Placement of Indoor Wireless Sensor Networks in Smart Buildings for IoT Applications

**DOI:** 10.3390/s20216212

**Published:** 2020-10-30

**Authors:** Mohammed A. Alanezi, Houssem R. E. H. Bouchekara, Muhammad S. Javaid

**Affiliations:** 1 Department of Computer Science and Engineering Technology, University of Hafr Al Batin, Hafr Al Batin 31991, Saudi Arabia; alanezi.mohd@uhb.edu.sa; 2 Department of Electrical Engineering, University of Hafr Al Batin, Hafr Al Batin 31991, Saudi Arabia; javaid@uhb.edu.sa

**Keywords:** IoT, router placement, sensor network, smart building

## Abstract

Internet of Things (IoT) is characterized by a system of interconnected devices capable of communicating with each other to carry out specific useful tasks. The connection between these devices is ensured by routers distributed in a network. Optimizing the placement of these routers in a distributed wireless sensor network (WSN) in a smart building is a tedious task. Computer-Aided Design (CAD) programs and software can simplify this task since they provide a robust and efficient tool. At the same time, experienced engineers from different backgrounds must play a prominent role in the abovementioned task. Therefore, specialized companies rely on both; a useful CAD tool along with the experience and the flair of a sound expert/engineer to optimally place routers in a WSN. This paper aims to develop a new approach based on the interaction between an efficient CAD tool and an experienced engineer for the optimal placement of routers in smart buildings for IoT applications. The approach follows a step-by-step procedure to weave an optimal network infrastructure, having both automatic and designer-intervention modes. Several case studies have been investigated, and the obtained results show that the developed approach produces a synthesized network with full coverage and a reduced number of routers.

## 1. Introduction

With the advent of low-power high-fidelity sensors capable of making precise measurements of the environmental parameters, the need to effectively link them in a network has arisen. Wireless communication networks have successfully replaced wired connections for efficient data transmission between sensor nodes, via radio communication. As a result, wireless sensor networks (WSN) have come into existence, and are playing an essential role in our daily lives [1]. WSNs also play a pivotal role in realizing the recent development of the Internet of Things (IoT), where the applications of such networks include security systems, environment monitoring, healthcare systems, and smart homes to name a few [2]. In one of the recent IoT applications, a WSN connects the legacy electrical equipment, incapable of communicating with standard protocols, with the smart grid controller [3]. The concept receives further attention from the research community when it is incorporated in an entire building to monitor and control appliances so that the building is automated. The purpose of this automation can be to make the building energy-efficient, or environmentally and inhabitant friendly. These automation systems are known as the building automation system (BAS). Energy efficiency is not only a focal point of research for large systems but also for systems as small as the sensors themselves [4,5]. Moreover, researchers are seeking adaptive solutions capable of providing more stable network performance with optimal network paths and resources [6].

Designing WSNs for the BAS has been a challenging task for the research community. Usually, while establishing an optimal arrangement of a network system, conflicting objective functions might arise [7]. A joint optimization model has been developed in [8] to optimize power, rate, and delay of radio sensor networks collectively. In [9], a large scale WSN is considered where authors utilize the genetic algorithm to determine the moving trajectory of the mobile sinks and an improved version of particle swarm optimization (PSO) to ascertain parking positions so that coverage rate is optimized. The same group developed the PSO-based coverage control algorithm in [10] considering both coverage rate and ensuring reduced energy consumption. On a smaller scale, an indoor localization of routers has been carried out in [11] using an interpolation algorithm and dual-frequency bands. For enhancing data collection rata, [12] presents a data-aggregation-based algorithm formulated as a linear programming problem. Energy-efficient software-defined WSNs are proposed by combining content awareness and adaptive data broadcast in [13], increasing the sensor’s lifespan. In [14,15], the authors utilized a CAD tool to model, simulate, and automate code generation for the optimal placement of routers in the design of WSN for BAS. Its extension was presented in [16], where the authors developed an interactive tool for network synthesis. The improved version of that in [16] claims to reduce design time while improving the quality of network topology. They offer a crude simulation-based trial-and-error approach to simulate multiple topologies one by one and select the better performing solution without the guarantee of optimality. It provides a graphical user interface (GUI) where a designer places the routers in a 2D floor plan to establish the connection between end devices (EDs)—which include sensors and actuators—and the base station (BS). This GUI provides a router placement plan for a single floor, considering that this is how building floors are distributed in rented residential units and offices. An exciting alternative was proposed in [17], where the duct system of a building is used as a waveguide to establish communication between various WSNs. The optimal placement of the sensor is not limited to BAS only. For example, in [18], a preferred placement of sensors to monitor human activity using smart textile systems and inertial measurement units, was presented.

Similarly, in [19,20], a GUI based interactive tool was developed for optimal placement of router nodes while considering different propagation models and terrain obstacles depicted in the floor plan. Reference [19] presents two methods to design the architecture of the WSN backbone: mixed integer linear programming (MILP) and the Dijkstras algorithm. The former one gives the exact solution to the problem for a network consisting of about 50 nodes in an hour. In contrast, the second method gives the suboptimal solution but was significantly faster than the first method. MILP, a computationally expensive method, was also utilized in [21] to design a WSN. In [22], the neural-gas algorithm was utilized to design the BAS, where it takes the information of building geometry, target constraints, and special zones and comes up with the candidate solutions for placing sensors in the network. Reference [22] differs from [16] in the sense that it determined the optimal placement of sensors, whereas [16] gave the optimal solution for a routers’ location that connects EDs with BS.

Another similar approach is developed in [23]. However, instead of the neural-gas algorithm, it used simulated annealing. It deployed a method of partitioning in which target space is subdivided into smaller sub-regions to deal with the dynamic environment of each sub-region. In this work, however, the target space is not explicitly divided, and the algorithm will automatically take care of the spatial variations. Search oriented strategies, primarily based on simulated annealing, were presented in [24]. It also considers the radiation pattern of antennae while determining the sensor positions. The algorithm looks for those positions of the nodes in the search space that satisfy the connectivity and coverage constraints. Instead of a WSN, Reference [25] used simulated annealing to find the optimal location of mesh routers in a wireless mesh network. In this paper, the underlying optimization algorithm remains the same, but the network has an additional gateway router to be considered while deploying communication protocols. The same objective was achieved in [26] using the firefly optimization algorithm, instead of simulated annealing. Other coverage constraints are beyond the scope of this work, as it is focused on ensuring the connectivity of fixed EDs to the BS. Another grid-based nodes deployment technique was presented in [27,28], to maximize the coverage of network sensors; however, the problem turns out to be constrained, in terms of being truly optimal, due to limited search space.

The main contributions of this paper are:

1. The development and deployment of a new technique that would result in a synthesis of backbone network architecture having a smaller number of routers in smaller amount of time, ensuring high fidelity connectivity between the EDs and the BS, throughout the target space.
2. Develop an interactive CAD based on a smart approach for optimal router placement.
3. Combine the benefits of the developed CAD and the experience of design engineers in an automatized manner.

The remainder of this paper is organized as follows. In Section 2, the proposed approach is presented and illustrated through a detailed example. The results are presented in Section 3. Finally, the main conclusions of the paper are drawn in Section 4.

## 2. Proposed Approach

### 2.1. Walkthrough

**Figure 1:**
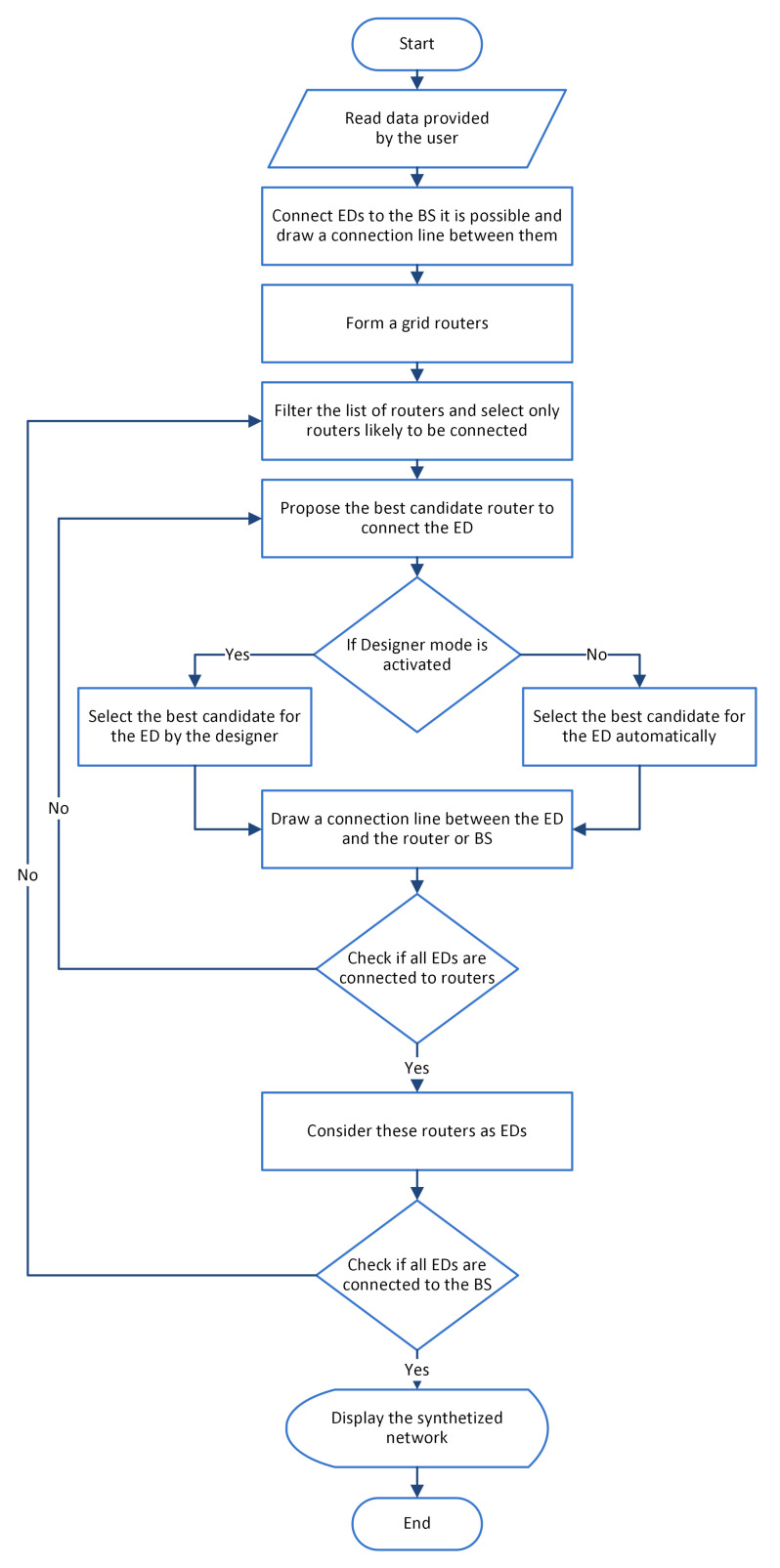
Flowchart of the proposed approach.

The proposed approach takes a step-by-step approach to develop the synthesized structure of routers. The flowchart of this approach is given in Figure 1. In the first step, the input data are provided by the user—the floor plan, the position of the BS, the Eds, and the thicknesses of the walls. Furthermore, communication parameters, like the operating frequency of the wireless sensors, are fed into the algorithm. 

The Free Space Path Loss (FSPL) in (dB) between two routers is given by:

(1) $$\mathrm{FSPL}=20\log d+20\log f+20\log\frac{4\pi }{c}-G_{t}-G_{r}$$

where d is the distance between the two routers [m], f is the frequency [GHz], c is the light speed [m/s], $G_{T}$ is the transmitter gain [dB] and $G_{r}$ is the receiver gain [dB].

In this paper, an operating frequency of 2.4 GHz is considered, $G_{t}$ and $G_{r}$ are supposed to be null, a −75 dB communication range (minimum FSPL) is assumed in a lossless free space medium. Equation (1) indicates that the assumed minimum FSPL is equivalent to 55 m as a maximal distance between two connected sensors (if there are no walls or obstacles in between). If there is an obstacle between the two sensors this 55 m distance is reduced in the function of the obstacle (or wall) thickness.

In the second step, a connection is established between the BS and EDs situated nearby. To be precise, EDs situated within a range of 55 m are linked to the BS, and a subsequent connection line—a thick black colored line—is then drawn to show that this ED has been connected. Later, in step 3, a grid of N × M potential routers is constructed over the entire floor plan. Routers placed on walls or obstacles are slightly moved to avoid the overlap.

In step 4, it was realized that not all routers can be connected to EDs. Therefore, the information of the routers that are likely to be connected to an ED (i.e., routers within 55 m range from EDs) are kept for the next step, and the remaining routers are removed, reducing the size of feed-forward matrices and consequently minimizing the processing time.

In the fifth step, the algorithm can either propose the best candidate router or automatically select the best candidate router for each ED, depending on the mode selected. The mechanism of selecting the best candidate router is the same as the one presented in [29]. In this mechanism, routers are sorted, and the ones with the ability to connect to more routers and are near to the BS are ranked highest. The first ranked router is the best candidate router. For example, if there are two routers where the first one can connect to two EDs while the second one can connect to three EDs, the second one is ranked better than the first one. This is the same if two routers have the same number of potential connections to EDs; however, if the first one is nearer to the BS then it is ranked better than the second one.

In the first mode of operation, the designer can either approve the algorithm proposal or select a different router based on his experience or any other external factor that the algorithm cannot take into consideration. A blue-colored connection line is drawn to show that this ED has been connected. This operation is repeated until all EDs are connected.

In step 6, the previously connected routers take the role of EDs, and the process from step 2 to step 5 is repeated until all EDs are fully connected to the BS via best candidate routers. In the end, the entire synthesized network is displayed.

### 2.2. Illustration with a Simple Network Setup

**Figure 2:**
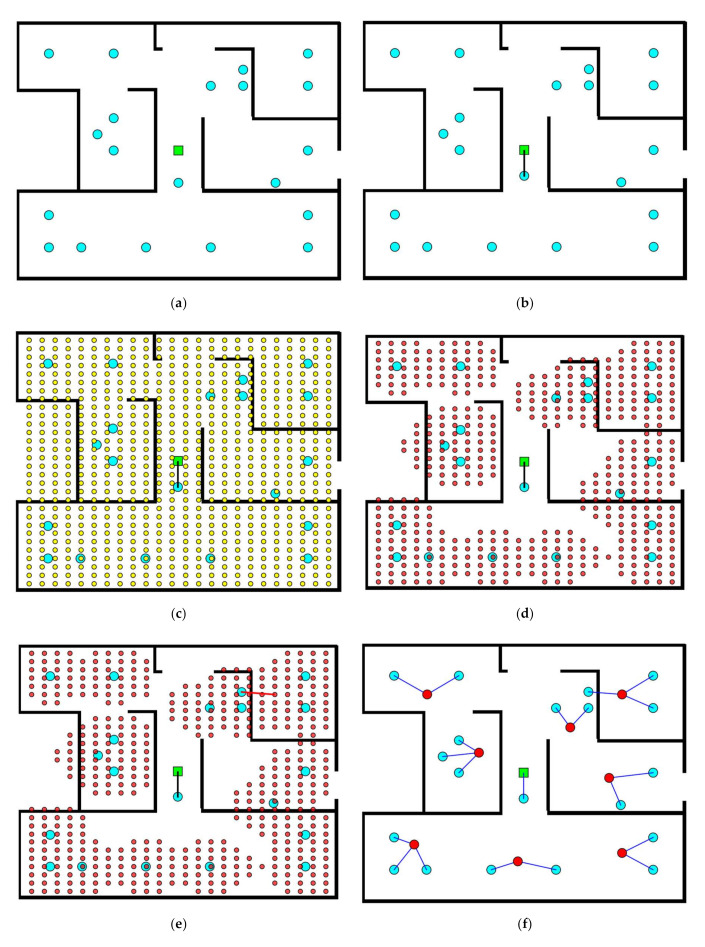

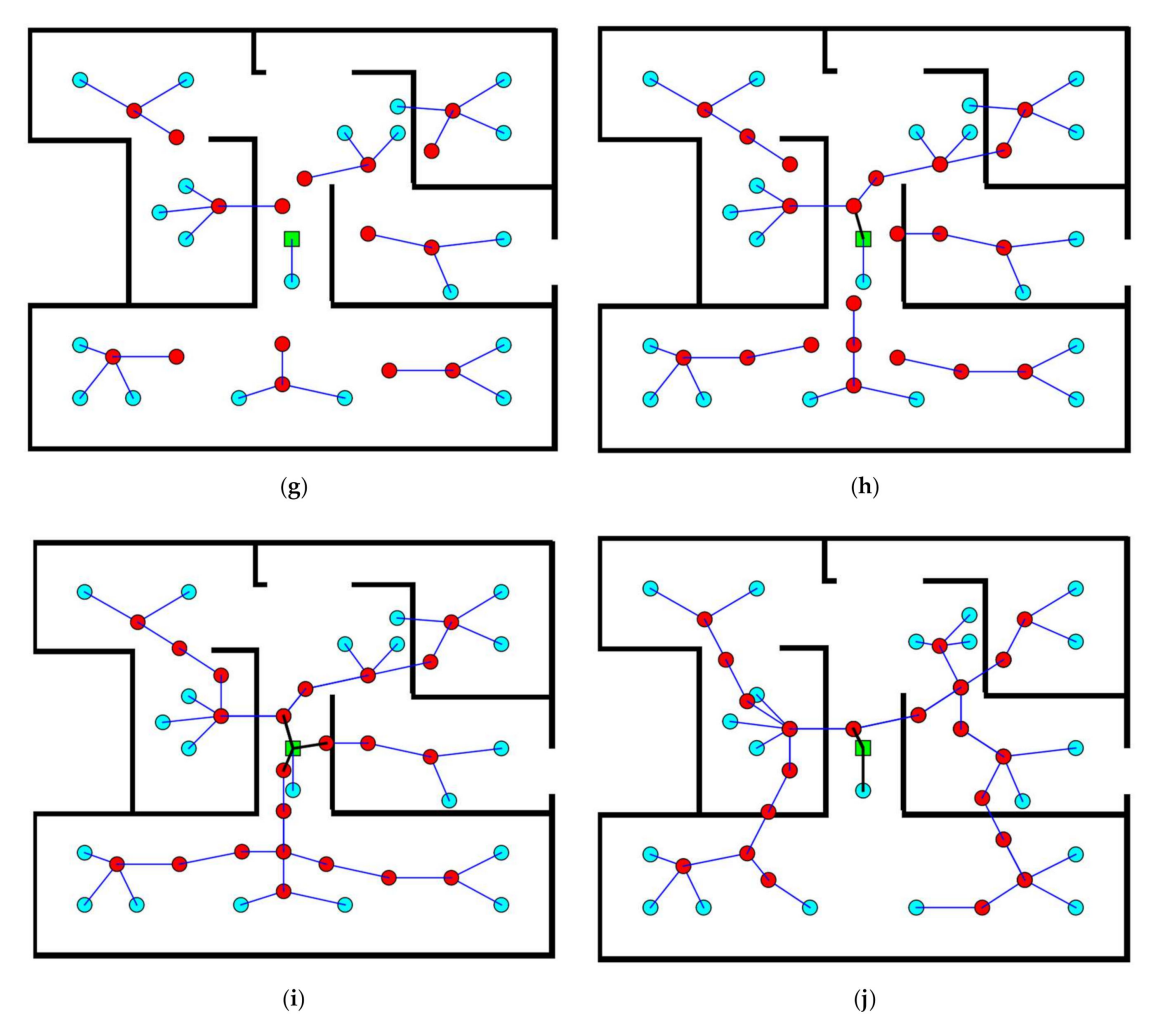
Illustrative example: (**a**) Initial layout; (**b**) Connection of an end device (ED) to the base station (BS); (**c**) grid construction over the entire floor plan; (**d**) routers list filtering; (**e**) best router proposal for a given ED; (**f**) connection of all EDs to routers; (**g**) connection of the first set of routers to a second set; (**h**) connection of the second set of routers to a third set; (**i**) final design; and (**j**) an alternative final design.

Step 1 deals with reading and storing all the data given by the user such as the dimensions of the layout, the obstacles, the EDs numbers and locations, and the BS location. In this illustrative example there are 19 EDs located at (50 m, 50 m), (150 m, 50 m), (150 m, 200 m), (400 m, 250 m), (450 m, 200 m), (50 m, 350 m), (100 m, 350 m), (200 m, 350 m), (450 m, 350 m), (450 m, 300 m), (300 m, 100 m), (350 m, 75 m), (150 m, 150 m), (350 m, 100 m), (300 m, 350 m), (50 m, 300 m), (125 m, 175 m), (450 m, 50 m), (450 m, 100 m) and (250 m, 240 m) and one BS located at (250 m, 200 m). The initial layout has a dimension of (500 m × 400 m), as shown in Figure 2a where EDs are represented by cyan colored circles whilst the BS is represented by a green-colored square. The origin (0 m, 0 m) is located in the upper left corner of the layout. Step 2 connects the EDs to the BS if they lie in close vicinity. In this example, there is one ED that can be connected directly to the BS because the distance between them is less than 55 m, as shown in Figure 2b. Consequently, in step 3, a grid of 24 × 30 = 720 potential routers is constructed over the entire floor plan, as shown in Figure 2c. Step 4 keeps only those routers that are likely to be connected to an ED and discards remaining routers from the list of potential routers, as shown in Figure 2d.

Assuming the selection of designer mode for step 5 of this illustration, the algorithm proposes the best candidate router to connect to each ED one by one. This choice is represented by a thick red line, as shown in Figure 2e. Whether the designer approves this choice or not, the whole process is repeated until all EDs are connected, as shown in Figure 2f where red circles represent connected routers.

Step 6 considers previously connected routers as EDs and the process from step 2 to step 5 is repeated until all the initial EDs are connected to the BS as shown in Figure 2g,h. Once all EDs are connected to the BS, the optimal network is obtained, as shown in Figure 2i. Based on designer choices, other networks can also be obtained. An alternative network choice is shown in Figure 2j. The first obtained design is composed of 22 routers, whilst the second one is composed of 21 routers.

## 3. Application and Results

The developed approach has been tested on different floor plans using a variety of dimensions and sizes and different numbers of EDs. The obtained networks have been compared with the synthesized networks using the CAD tool developed in [29].

### 3.1. Case Study 1

**Figure 3:**
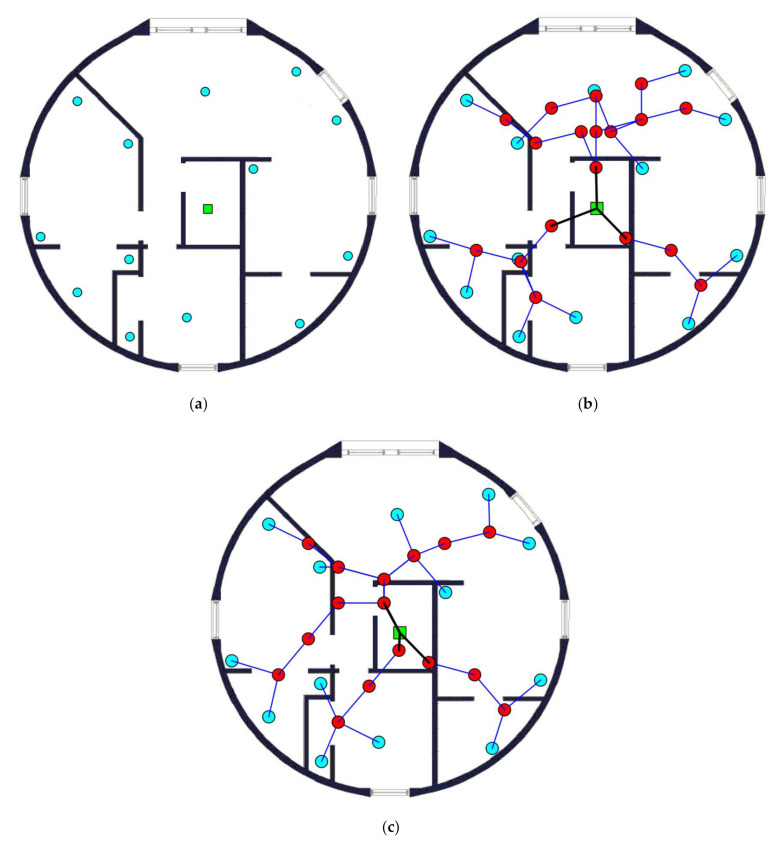
Case study 1: (**a**) Initial layout; (**b**) synthesized network using the CAD tool; and (**c**) synthesized network using the proposed approach.

The initial layout (430 m × 420 m) for this case is given in Figure 3a. There are 13 EDs and 1 BS. The synthesized network using the fully automatized CAD tool is given in Figure 3b while the one obtained using the proposed approach is given in Figure 3c. It can be seen from these figures that, for the first network, there are 18 placed routers whilst for the second network, there are 16 placed routers. It can also be noticed that the synthesized network using the proposed approach is much more optimized than the one using the CAD tool without the interaction of the expert designer.

The detailed information about the synthesized network is tabulated in Table 1. In this table, the first column represents the node BS, ED or router number, the second column shown the type of the node where ‘1’ stands for BS node, ‘2’ stands for ED, ‘3’ stands for the router, and the last two columns represent the x-coordinate and y-coordinate of each node, respectively.

### 3.2. Case Study 2

**Figure 4:**
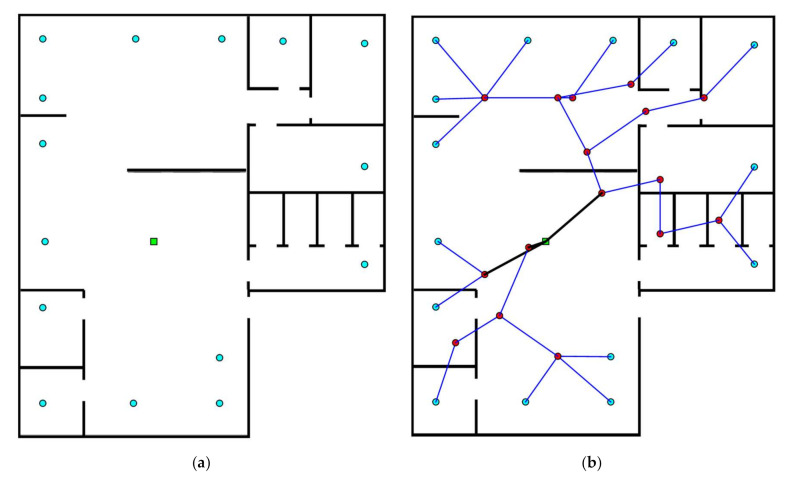

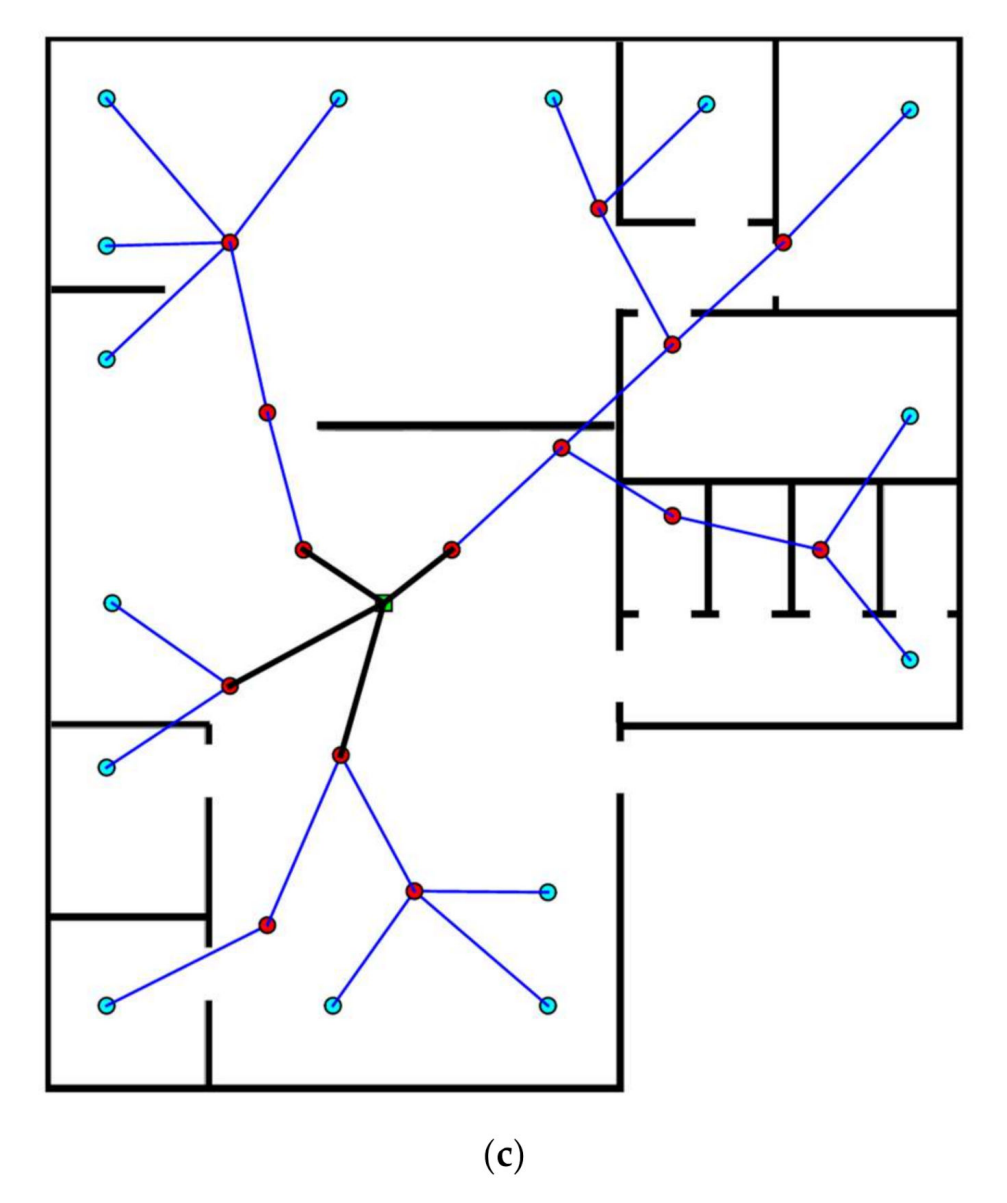
Case study 2: (**a**) Initial layout; (**b**) synthesized network using the CAD tool; and (**c**) synthesized network using the proposed approach.

For this case study, the initial layout has the dimensions of (310 m × 270 m), as shown in Figure 4a. The area is equipped with 15 EDs and 1 BS. Figure 4b shows the synthesized network obtained using a fully automatized CAD tool, while Figure 4c displays the one obtained using the proposed approach. With a fully automized CAD tool, 16 routers are placed, whereas the proposed algorithm offers the solution by optimally placing only 14 routers. In congruence with the previous case study, the synthesized network using the proposed approach produces better results than those using the CAD tool only. The detailed information about the synthesized network obtained using the proposed approach is tabulated in Table 2.

### 3.3. Case Study 3

**Figure 5:**
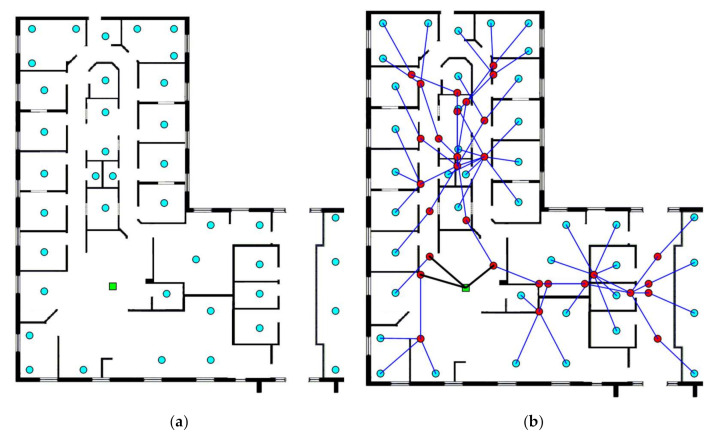

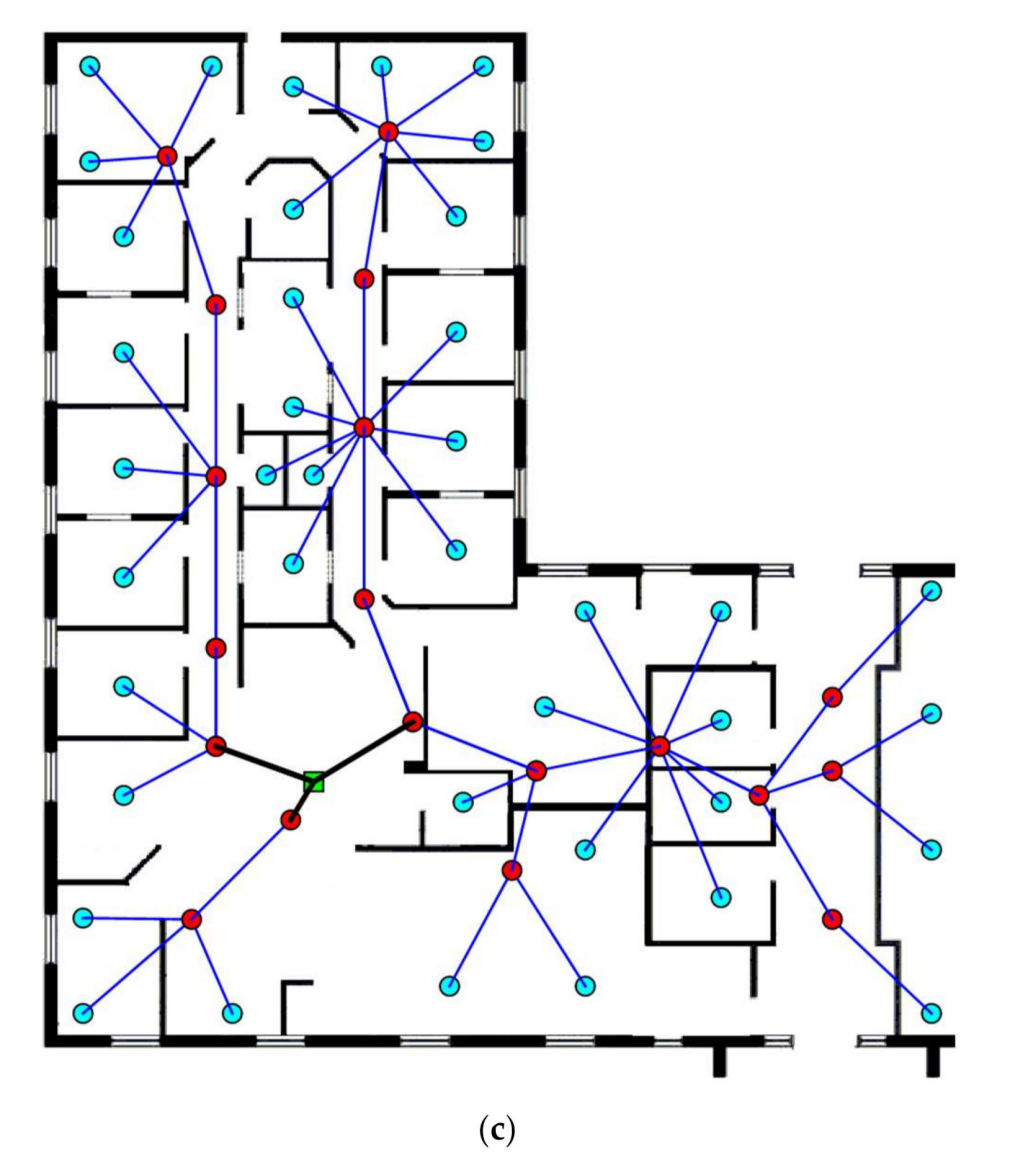
Case study 3: (**a**) Initial layout; (**b**) synthesized network using the CAD tool; and (**c**) synthesized network using the proposed approach.

In case three, the layout dimensions are (380 m × 340 m), the number of EDs are 40 with 1 BS, as shown in Figure 5a. Without expert intervention, i.e., only using the CAD, the network architecture obtained is shown in Figure 5b, whereas, the one obtained using the proposed approach is depicted in Figure 5c. The proposed technique provides the solution with 19 routers in comparison with 30 routers which were obtained using the CAD only approach. Thus, an optimal result is obtained using the proposed approach. Table 3 tabulates the necessary information related to the coordinates and the position of every node in the network.

## 4. Conclusions

In this paper, a new efficient and interactive synthesis algorithm for optimal router placement in WSNs has been proposed, developed, and implemented. This algorithm has been first illustrated using a simple network setup. Then, three case studies, with different difficulties and configurations, were investigated to test the validity of the proposed algorithm. Finally, a case study at the University of Hafr Al Batin was investigated. The obtained results are satisfactory compared to the use of CAD only. The interaction between the designer and the CAD led to better results.

However, there are some perspectives to investigate in future studies. One aspect could be the design of WSNs with some redundancy to avoid network failure that can be caused by nodes failures. Another future axis could be to include more details about the building, such as the types of materials used for walls and windows. Finally, considering more than one base station could also be investigated in our future work.

## Figures and Tables

**Table 1:** Nodes of the synthesized network using the proposed approach for case study 1.

Node #	Node Type	x	y
1	1	225	225
2	2	76	101
3	2	34	257
4	2	134	150
5	2	135	283
6	2	76	321
7	2	136	372
8	2	201	350
9	2	330	357
10	2	385	279
11	2	277	179
12	2	326	67
13	2	222	90
14	2	372	123
15	3	87	273
16	3	121	123
17	3	121	232
18	3	155	150
19	3	155	191
20	3	155	327
21	3	190	286
22	3	207	164
23	3	207	191
24	3	224	245
25	3	241	137
26	3	258	259
27	3	276	123
28	3	310	273
29	3	327	110
30	3	344	313

**Table 2:** Nodes of the synthesized network using the proposed approach for case study 2.

Node #	Node Type	x	y
1	1	75	75
2	2	18	215
3	2	18	285
4	2	85	285
5	2	148	285
6	2	148	252
7	2	255	183
8	2	255	112
9	2	255	22
10	2	195	20
11	2	150	18
12	2	87	18
13	2	18	18
14	2	18	62
15	2	18	95
16	2	20	167
17	3	55	61
18	3	55	191
19	3	66	111
20	3	66	261
21	3	76	151
22	3	87	211
23	3	109	251
24	3	120	151
25	3	152	121
26	3	163	51
27	3	185	91
28	3	185	141
29	3	218	61
30	3	229	151

**Table 3:** Nodes of the synthesized network using the proposed approach for case study 3.

Node #	Node Type	x	y
1	1	100	275
2	2	18	13
3	2	63	13
4	2	18	48
5	2	30	75
6	2	30	118
7	2	30	160
8	2	30	200
9	2	30	240
10	2	30	280
11	2	15	325
12	2	15	360
13	2	70	360
14	2	328	360
15	2	328	300
16	2	328	250
17	2	328	205
18	2	250	318
19	2	250	283
20	2	250	253
21	2	155	283
22	2	125	13
23	2	163	13
24	2	163	40
25	2	153	68
26	2	153	110
27	2	153	150
28	2	153	190
29	2	93	20
30	2	93	65
31	2	93	98
32	2	93	138
33	2	83	163
34	2	100	163
35	2	93	195
36	2	185	248
37	2	200	300
38	2	200	350
39	2	150	350
40	2	250	213
41	2	200	213
42	3	46	46
43	3	55	326
44	3	64	100
45	3	64	163
46	3	64	226
47	3	64	262
48	3	92	289
49	3	119	91
50	3	119	145
51	3	119	208
52	3	128	37
53	3	137	253
54	3	173	308
55	3	182	271
56	3	228	262
57	3	264	280
58	3	291	244
59	3	291	271
60	3	291	326

## Abbreviations

BS	Base Station
BAS	Building Automation System
CAD	Computer-Aided Design
EDs	End Devices
GUI	Graphical User Interface
IoT	Internet of Things
MILP	Mixed Integer Linear Programming
WSN	Wireless Sensor Network
